# Utilization of Electrodeionization for Lithium Removal

**DOI:** 10.1021/acsomega.2c08095

**Published:** 2023-05-10

**Authors:** Gülseren Demir, Ayşe Nur Mert, Özgür Arar

**Affiliations:** Chemistry Department, Faculty of Science, Ege University, Izmir 35040, Türkiye

## Abstract

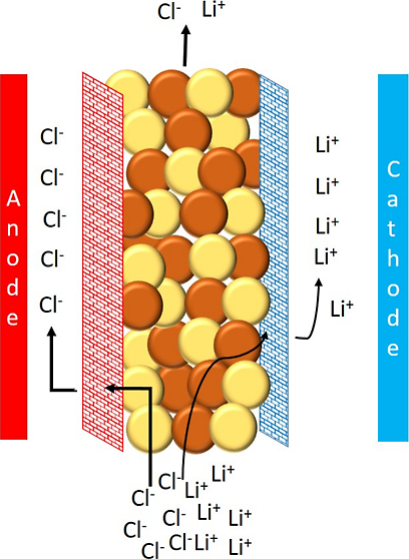

In this work, usage of a hybrid polymeric ion exchange resin and a polymeric ion exchange membrane in the same unit to remove Li^+^ from aqueous solutions was reported. The effects of the applied potential difference to the electrodes, the flow rate of the Li-containing solution, the presence of coexisting ions (Na^+^, K^+^, Ca^2+^, Ba^2+^, and Mg^2+^), and the influence of the electrolyte concentration in the anode and cathode chambers on Li^+^ removal were investigated. At 20 V, 99% of Li^+^ was removed from the Li-containing solution. In addition, a decrease in the flow rate of the Li-containing solution from 2 to 1 L/h resulted in a decrease in the removal rate from 99 to 94%. Similar results were obtained when the concentration of Na_2_SO_4_ was decreased from 0.01 to 0.005 M. The selectivity test showed that the simultaneous presence of monovalent ions such as Na^+^ and K^+^ did not change the removal rate of Li^+^. However, the presence of divalent ions, Ca^2+^, Mg^2+^, and Ba^2+^, reduced the removal rate of Li^+^. Under optimal conditions, the mass transport coefficient of Li^+^ was found as 5.39 × 10^–4^ m/s, and the specific energy consumption was found as 106.2 W h/g LiCl. Electrodeionization provided stable performance in terms of the removal rate and transport of Li^+^ from the central compartment to the cathode compartment.

## 1 Introduction

Because of its high energy density and electrochemical potential, lithium is used in many kinds of applications, especially in lithium-ion batteries.^1^ Lithium is also used in ceramics and glass, air conditioning systems, and the treatment of bipolar disorder.^2−5^ Because of the increasing use of lithium-ion batteries in various fields, the consumption of lithium has increased, so the recovery and extraction of Li^+^ from aqueous samples have gained traction.^6,7^ It has also been shown that extracting lithium from various saline waters, such as geothermal water and seawater, which are known to be significant sources of lithium, is less costly than extracting lithium from rocks through mining.^2−4,8^

To date, various methods have been used to remove/recover Li^+^ from various water samples. Such methods can be divided into sorption,^9,10^ biosorption,^2,4,11^ ion exchange,^12,13^ solvent extraction,^14,15^ precipitation,^16^ and membrane processes.^7,17−20^

Electrodeionization (EDI) is an electromembrane technique that removes ionic and weak electrolyte species using an electrical potential difference as the driving force.^21−23^ EDI has been used to remove impurities, purify mother liquor, and produce ultrapure water. Review articles summarize these applications, advantages, and limitations of EDI and electromembrane processes.^22,24−30^

To this day, electromembrane processes have been used in various ways to remove/purify solutions containing Li^+^. Bajestani et al. (2020) prepared a lithium-selective cation exchange membrane (CEM) and used it in the electrodialysis (ED) system to recover Li^+^ from a bromide solution by ED. For this purpose, the surface of the CEM was coated with a Li^+^-selective adsorbent of LiCo_0.5_Mn_1.5_O_4_. The authors reported that the prepared membrane exhibited superior Li^+^ selectivity compared to commercially available membranes.^31^ In another work, Tsuyoshi Hoshino (2013) developed a 2-step ED process to recover Li^+^ from seawater. In the first step of the work, a monovalent selective Selemion CSO CEM was used in an ED cell to remove divalent cations from seawater. In the second step of the work, the ionic liquid *N*,*N*,*N*-trimethyl-*N*-propylammonium bis(trifluoromethanesulfonyl)imide, which has low Li^+^ conductivity, was used in the ED cell. The seawater treated in the first stage was fed into the ED cell, and Na^+^ and K^+^ in the seawater could pass through the ionic liquid chamber, but Li^+^ could not pass through this chamber. At the end of the experiment, a Li^+^-rich solution was obtained.^32^

Melnikov et al. applied combined electromembrane processes, namely, ED reversal (EDR), ED, and EDI, to recover lithium chloride-containing process solutions containing dimethylacetamide, isobutyl alcohol, water, lithium chloride, and some other ionic species and also to purify the organic solvents. In the first phase of the work, EDR was used to prepare lithium hydroxide and neutralize the process solution. In the second phase of the work, ED was used to demineralize the process solution, and ED was continued until the LiCl concentration reached 0.05%. In the last phase, EDI was used to achieve complete removal of ionic impurities from the organic solvent. The LiCl content was 0.84% at the beginning of the electromembrane process and was decreased to 0.07% at the end of the ED process. After passing through the EDI cell, the Li content was back to 0%.^33^

In this study, an EDI cell was constructed and used to eliminate Li^+^ from aqueous solutions. The effects of the potential applied to the electrodes; the stream rate of the Li-containing solution; the presence of Na^+^, K^+^, Ca^2+^, Mg^2+^, and Ba^2+^ ions; and the impact of the electrolyte concentration in the anode and cathode chambers on Li^+^ elimination were examined.

## 2 Results and Discussion

### 2.1 Effect of Applied Potential Difference on Li^+^ Elimination

**Figure 1:**
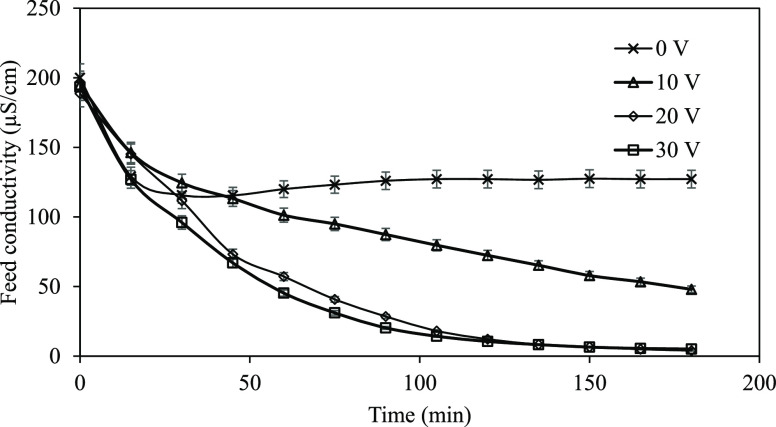
Variation in the conductivity of a Li^+^-containing solution in relation to the applied potential difference over time (Li^+^ concentration: 5 mg/L, feed flow rate: 2 L/h, Na_2_SO_4_ concentration: 0.01 M, and Na_2_SO_4_ flow rate: 6 L/h).

In electromembrane processes, the potential applied to the electrode stacks transports the ions in the solution through the ion exchange resins and membranes to the anode or cathode.^34,35^ Therefore, it should be optimized to find a suitable potential for ion removal. For this purpose, the applied potential changed in the range of 10–30 V. The change in the conductivity of the solution containing Li^+^ versus time at different applied potentials is shown in Figure 1.

From Figure 1, it can be seen that the application of the potential causes the migration of ions into the anode and cathode chambers so that the ion concentration in the feed solution decreases, and so does the conductivity of the solution. Figure 1 also demonstrates that the conductivity of the solution decreased from 195 to 48 μS/cm when a voltage of 10 V was supplied to the EDI cell. However, when 20 and 30 V were applied to the system, the solution’s conductivity dropped to 5 μS/cm. As explained earlier, the increase in driving force resulted in more ions being transported, and therefore, the solution was less conductive. The same experiments were performed without applying the potential to the EDI stack (given as 0 V). The results revealed that the conductivity of the feed solution decreased from 200 to 127 μS/cm. The diffusion of H^+^ ions causes a decrease in the conductivity of the solution from the dilute compartment to the cathode compartment.

**Figure 2:**
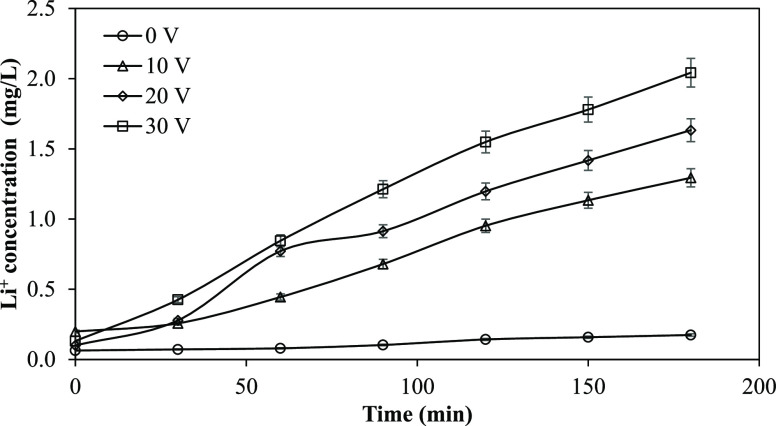
Li^+^ concentration in the cathode compartment vs time (Li^+^ concentration: 5 mg/L, feed flow rate: 2 L/h, Na_2_SO_4_ concentration: 0.01 M, and Na_2_SO_4_ flow rate: 6 L/h).

The variation of the Li^+^ concentration in the cathode compartment as a function of applied potential at different intervals is shown in Figure 2.

**Figure 3:**
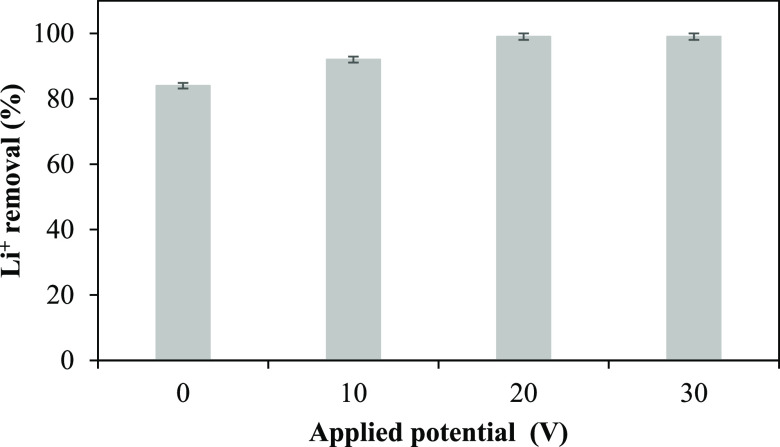
Li^+^ removal as a function of the potential difference applied to electrodes (Li^+^ concentration: 5 mg/L, feed flow rate: 2 L/h, Na_2_SO_4_ concentration: 0.01 M, and Na_2_SO_4_ flow rate: 6 L/h).

Figure 2 shows that the transport of Li^+^ from the dilute chamber to the cathode chamber increases with time. In addition, the conveyance of Li^+^ to the cathode chamber was enhanced by increasing the applied potential. The 26% of Li^+^ transferred to the cathode at 10 V was increased to 32% by increasing the applied potential up to 20 V, and at 30 V, the transport rate was 40%. Water transfer is associated with the exchange of counterions. Ion exchangers, therefore, prefer ions that have a smaller solvated size. Li^+^ ions have a poor exchange rate with strongly acidic cation ion exchange resin carrying sulfonic acid groups,^36^ and this property could enhance the transport of ions to the cathode compartment. Without applying the potential to the EDI stack, the transport of Li^+^ from the dilute compartment to the cathode compartment also occurs by diffusion. However, the Li^+^ concentration in the cathode compartment is about 0.2 mg/L, so only a small amount of Li^+^ is transferred to the cathode compartment compared to migration. The change in the removal of Li^+^ versus the applied potential is demonstrated in Figure 3.

When a voltage of 10 V was supplied to the system, 92% of Li^+^ in the solution was removed. When 20 and 30 V were applied to the stack, 99% of Li^+^ was removed from the solution. 84% of Li^+^ was removed from the solution without applying a potential. Applying the potential to the EDI stack enhanced the removal rate and, as shown in Figure 3, the transport of Li^+^ from the dilute compartment to the cathode compartment.

Various researchers have also reported a similar finding. Rathi and Kumar (2022) applied EDI to the removal of As(V) and reported that the removal of As(V) increased with the increase of the applied potential. At an applied potential of 20 V, almost 100% of As(V) was removed from the solution.^37^ In another work, Zahakifar et al. (2020) used EDI to remove Th(IV) from aqueous solutions and reported that the performance of EDI was enhanced when the voltage was higher than 10 V.^38^

The removal rate of Li^+^ was the same at 20 and 30 V. In order to perform experiments with lower power consumption, 20 V was chosen as the optimum potential and used in further experiments.

### 2.2 Impact of the Feed Flow Rate on Li^+^ Removal

**Figure 4:**
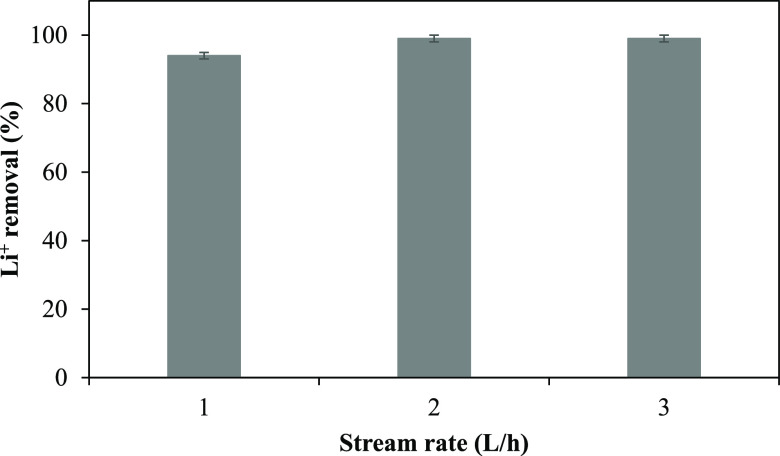
Removal of Li^+^ at different feed flow rates (applied voltage: 20 V Li^+^, concentration: 5 mg/L, Na_2_SO_4_ concentration: 0.01 M, and Na_2_SO_4_ flow rate: 6 L/h).

The transport of ions is proportional to the residence time in the electroactive media. Its migration from the solution to the electrode compartment consists of several diffusion/migration steps, such as from the solution resin surface, from the resin surface to inside the resin particle, etc.^25,39^ To investigate the effect of the feed flow rate on Li^+^ removal, 5 mg/L containing solution was circulated in the dilute compartment at different flow rates (1, 2, and 3 L/h). The change in Li^+^ removal rate versus time is depicted in Figure 4.

When the solution containing Li^+^ was recycled at 1 L/h, 94% of Li^+^ was eliminated. Expanding the stream rate to 2 L/h increased the elimination of Li^+^ to 99%, but increasing the stream rate to 3 L/h did not affect the rate of Li^+^ removal.

The tests were performed in the batch mode; i.e., the same solution was circulated in the central chamber for 3 h. When the stream rate was expanded, the same solution circulated much longer within the diluted chamber, which could increase the elimination rate of Li. The feed solution’s conductivity at the end of the 3 h operation at a flow rate of 1 L/h was about 15 μS/cm. At a flow rate of 2 and 3 L/h, the conductivity of the feed decreased to 5 μS/cm.

Similar results were also observed in previous reports. Zhang and Chen (2016) used the EDI technique to remove NO_3_^–^ ions from aqueous solutions. The authors of that study found that the feed flow rate affected the removal rate. At a feed rate of 6 L/h, less than 90% of NO_3_^–^ was removed from the solution, but increasing the flow rate from 6 to 9 L/h improved the removal rate by more than 95%.^40^ Zhang et al. (2014) removed Cs^+^ ions using EDI in another work. The authors found that increasing the flow rate from 2 to 6 L/h improved the removal efficiency, but further increasing the flow rate to 10 L/h reduced the removal rate of Cs^+^.^41^

The 2 L/h flow rate was chosen as an optimum and used in subsequent experiments.

### 2.3 Effect of the Na_2_SO_4_ Concentration in the Electrode Compartment on Li^+^ Elimination

The current delivered to the electrodes is passed through the electrolyte in the electrode chambers. It establishes an electrical connection between the electrodes with the help of the ion exchange resins and the solution circulating in the central chamber. The composition and concentration of the electrolyte in the electrode compartment affect the EDI efficiency. For example, Feng et al. reported that when pure water circulated in the electrode compartment, no ions were present to carry the current, so the migration of Ni^2+^ to the cathode compartment could not be observed. However, the addition of Na_2_SO_4_ generated ions in the solutions, and the ions carried the current, and Ni^2+^ was transported to the cathode compartment.^42^ In another work, Bouhidel and Lakehal used amphoteric NH_4_CH_3_COO in the electrode compartment, and they observed that using NH_4_CH_3_COO instead of NaCl increased efficiency.^43^

**Figure 5:**
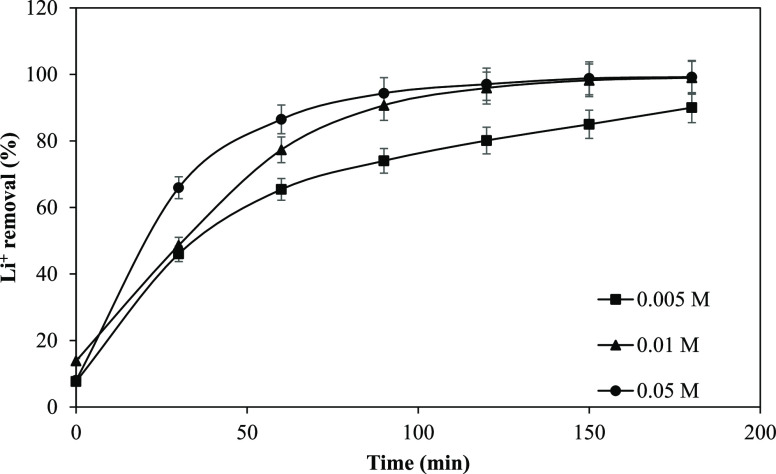
Removal of Li^+^ vs time as a function of the Na_2_SO_4_ concentration (applied voltage: 20 V, feed flow rate: 2 L/h, Li^+^ concentration: 5 mg/L, and Na_2_SO_4_ flow rate: 6 L/h).

In our case, we used a Na_2_SO_4_ solution at different concentrations. The elimination of Li^+^ at various Na_2_SO_4_ concentrations is depicted in Figure 5.

**Figure 6:**
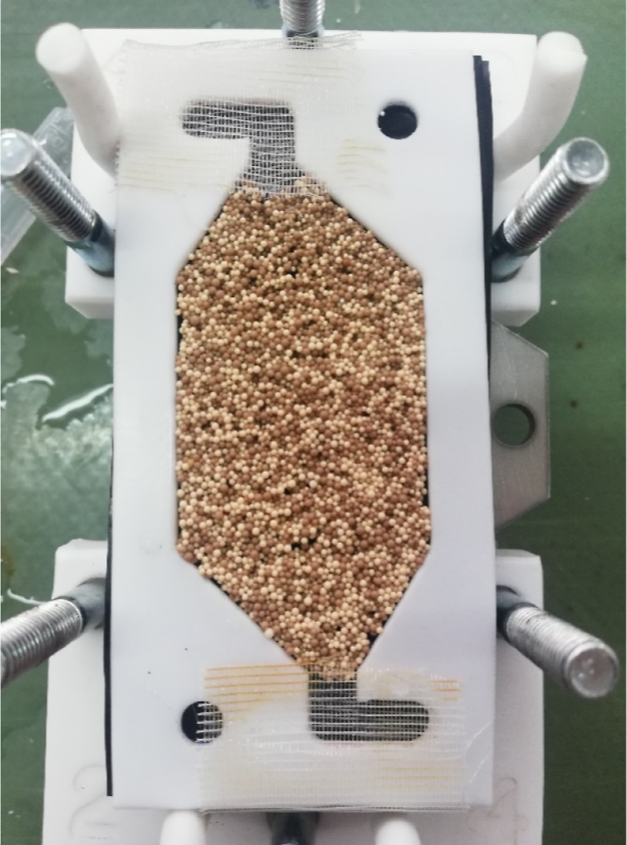
Mixed-bed resin-filled central compartment.

When the 0.005 M Na_2_SO_4_ solution circulated in the electrode compartment, 90% of Li^+^ was removed. Increasing the Na_2_SO_4_ concentration from 0.005 to 0.01 M enhanced the removal rate up to 99%. Figure 6 also shows that when the Na_2_SO_4_ concentration in the solution was changed from 0.005 M to 0.05 M in the first 90 min, the removal of Li^+^ was higher than that at 0.005 and 0.01 M. Increasing the electrolyte concentration in the electrode compartment leads to an increase in the electrical current of the EDI,^44^ which accelerates ion transport.

### 2.4 Effect of Coexisting Ions on the Removal of Li^+^

More than 80% of the global continent’s lithium deposits are in salt lake brines,^45^ and certainly, many ions are present in the same water samples. The presence of such ions may affect the transport of Li^+^ ions through ion exchange resins and membranes. To clarify the influence of the other ions in the water, a selectivity test was performed. The presence of Na^+^, K^+^, Ca^2+^, Mg^2+^, and Ba^2+^ in the removal of Li^+^ is discussed in this section. The concentration of coexisting ions and Li^+^ in the solution was set at 5 mg/L. The flow rate was set to 2 L/h, and 20 V was applied to the EDI stack. The variation of Li^+^ removal in the coexistence of different ions is shown in Table 1. The results show that the presence of Na^+^ and K^+^ at low concentrations had no effect on the removal of Li^+^, and 99% of Li^+^ was removed from the solution; on the other hand, the presence of divalent ions Ca^2+^, Mg^2+^, and Ba^2+^ reduced the removal rate of Li^+^ ions.

**Table 1:** Effect of the Coexisting Ion on the Removal of Li^+^

ion in the feed solution	ion concentration (mg/L)	removal rate (%)	selectivity
Li^+^	5.2	99	1.01
Na^+^	5.0	97	
Li^+^	5.0	99	1.01
K^+^	5.3	99	
Li^+^	5.5	96	1.01
Ca^2+^	5.2	98	
Li^+^	5.8	96	0.96
Mg^2+^	6.0	99	
Li^+^	5.1	93	0.95
Ba^2+^	4.7	99	

The selectivity (α) of Li^+^ in the presence of coexisting ions was determined from the separation coefficient (α) and calculated using the following equation (eq 1) where *C*_Li_^I^ is the initial concentration of the Li^+^ ion, *C*_Li_^F^ is the final concentration of the Li^+^ ion in the diluted solution, *C*_co_^I^ is the initial concentration of the coexisting ion in the solution, and *C*_co_^F^ is the final concentration of the coexisting ion in the diluted solution.^46,47^ Results are represented in Table 1.
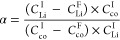
1

When α-values are greater than 1, this indicates preferential transport of Li^+^ ions, and when α is less than 1, this indicates preferential transport of coexisting ions.^48^ The transport of Li^+^ was favored by the presence of K^+^ and Na^+^ ions, and in the presence of divalent cations such as Ca^2+^, Mg^2+^, and Ba^2+^, the removal rate of Li^+^ decreased. This can be explained as follows: the migration flux of species *i* is defined in the following equation (eq 2).^49^
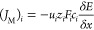
2where *u*_*i*_ is the mobility of species *i* in the pore fluid (m^2^ s^–1^ V^–1^), *z*_*i*_ is the electrical charge of species *i*, *F* is the Faraday constant (9.65 × 10^4^ C/mol), and *E* is the electrical potential (V).

From eq 2, it can be seen that the migration flux is linearly proportional to the ionic charge. Since the charge of divalent cations is greater than that of Li^+^ ions, the current is preferentially carried by these cations, so the removal rate of Li^+^ decreases.

The results obtained were compared with other membrane processes for the removal of Li^+^, and the results are summarized in Table 2.

**Table 2:** Removal of Li^+^ by Different Membrane Processes

membrane processes	experimental conditions	removal/recovery of Li (%)	reference
reverse osmosis	cell: SEPA CF-HP, membrane: FilmTec FT-30, Li^+^ concentration: ≈250 mg/L, pressure: 27.6 bars	>98	(50)
polymer-enhanced ultrafiltration	polymer: poly(potassium sulfopropyl acrylate) polymer: Li^+^ molar ratio of 20:1, pH: 5.7, pressure: 2.0 bar, Li^+^ concentration: 42.4 mg/L	>80	(51)
electrodialysis	membranes: ASTOM CIMS and ACS, applied voltage: 5 V, linear flow velocity: 6.2 cm/s, Li^+^ concentration: 140 mg/L, operation time: 2 h	72	(52)
electrodialysis	pilot scale ED stack: Selemion CSO and Selemion ASA membranes, operation time: 3 h, current density: 5.9 A/m^2^, feed linear velocity: 7.1 cm/s, Li^+^ concentration: 150 mg/L	94.5	(53)
electrodeionization	micro-flow cell EDI system: Selemion AHT and CMD membrane, applied voltage: 20 V, feed flow rate: 2 L/h, operation time: 3 h, Purolite C145 + A500 plus in mixed-bed configuration, Li^+^ concentration: 5 mg/L	99	this work

As shown in Table 2, various membrane processes can be used to remove and recover Li^+^ ions. The efficiency of such membrane processes is determined by the composition of the solution, the number of cells in the ED stack, the potential difference applied to the electrodes, and other factors. The findings of this study indicate that EDI can be used as an alternative membrane technique for the removal/recovery of Li^+^ from water samples.

### 2.5 Evaluation of EDI Performance

The performance of the EDI in terms of the mass transfer coefficient (*k*) and flux (*j*), current efficiency (CE; %), and the specific power consumption (SPC) for Li^+^ transport is calculated using the corresponding formulas^42,54−57^ given in the following equations (eqs 3–6), and results are summarized in Table 3.
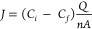
3
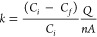
4
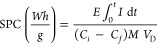
5
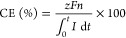
6where *C*_*i*_ is the initial lithium concentration in the feed solution (mol/m^3^), *C*_*f*_ is final lithium concentration in the feed solution (mol/m^3^), *Q* is the feed flow rate (m^3^/s), *n* is the number of cell pairs (1, in our case), *A* is the membrane area (m^2^), *M* is the molar mass of LiCl (42.394 g/mol), *V*_D_ is the volume of the solution which was fed to the central compartment (in our case, 0.001 m^3^), *z* is the valency of the ion (1 for Li^+^), *F* is the Faraday constant (96 500 C/mol), and *n* is the mole number of the Li^+^ ion removed (mol).

**Table 3:** Computed Numerical Values for Li^+^ Removal

	applied voltage (V)			flow rate (L/h)			Na_2_SO_4_ concentration (M)			Selectivity				
calculated value	10	20	30	1	2	3	0.005	0.01	0.05	Na	K	Ca	Mg	Ba
*k* (m/s)	4.99 × 10^–4^	5.39 × 10^–4^	5.37 × 10^–4^	5.12 × 10^–4^	5.39 × 10^–4^	5.38 × 10^–4^	4.90 × 10^–4^	5.39 × 10^–4^	5.40 × 10^–4^	5.32 × 10^–4^	5.40 × 10^–4^	5.23 × 10^–4^	5.21 × 10^–4^	5.09 × 10^–4^
*j* (mol/m^2^ s^1^)	3.82 × 10^–4^	3.69 × 10^–4^	3.68 × 10^–4^	3.84 × 10^–4^	3.69 × 10^–4^	4.03 × 10^–4^	3.53 × 10^–4^	3.69 × 10^–4^	3.98 × 10^–4^	3.95 × 10^–4^	4.14 × 10^–4^	4.14 × 10^–4^	4.36 × 10^–4^	3.77 × 10^–4^
SPC (W h/g LiCl)	20.59	106.2	234.49	117.17	106.2	94.05	62.7	106.2	75.89	98.46	69.90	86.99	111.90	70.78
CE (%)	31	12	8	11	12	13	20	12	17	13	18	15	11	18

The results showed that augmentation of the potential from 10 to 20 volts improved the mass transfer coefficient of Li^+^. However, a further increase from 20 to 30 V brought little change in the *k* value. Similar trends were also observed for different flow rates and Na_2_SO_4_ concentrations. Increasing the flow rate from 1 to 2 L/h (or the Na_2_SO_4_ concentration from 0.005 to 0.01 M) improved the *k* value, but the further increase did not improve the *k* value. The *k*-value is proportional to the initial and final concentrations of the ions. The initial and final concentrations of Li^+^ ions were very close at a flow rate of 2 and 3 L/h, so the calculated *k* values were the same.

The mass transfer coefficient was also calculated for different ions and was 1.56 × 10^–5^ for thorium (IV),^38^ 2.5 × 10^–6^ for boron,^47^ 5.42 × 10^–4^ for Mn^2+^,^58^ 1.8 × 10^–6^ for silica,^47^ 1.25 × 10^–5^ for the Cu^2+^ ion,^59^ and 1.5 × 10^–5^ for the Ni^2+^ ion.^60^ The calculated *k* values of Li^+^ ions are close to those of the Mn^2+^ ions and are larger than those of the other ions. This difference is due to the different experimental conditions, the resin used in the EDI stack, and the properties of the ions, such as the charge and hydrogenated radiation of the ion and the resin’s affinity for the ion.

When the applied voltage was raised, the SPC values rose. The SPC value is linearly proportional to the applied potential, so an increase in the potential that has been used leads to a rise in the SPC value.

An increase in applied potential resulted in a decrease in current efficiency. This could be due to the fact that most of the applied potential can be used for water splitting and not for ion transport.^61^ Change in the feed flow rate did not create a notable effect on current efficiency and was about 10%. The presence of interfering ions influenced the current efficiency. The presence of the co-existing ion change resin phase in the removal stage and such change in resin composition can change current efficiency.^62^

The calculated SPC values were compared with those of previously published work. Liu et al. used a liquid membrane ED system to extract Li^+^ from sols with high Mg/Li ratios and showed the lowest energy consumption 16 W h/g Li at a current density of 4.375 A/m^–2^.^63^ In another work, Nie et al. separated lithium ions from magnesium ions. They separated lithium ions from magnesium ions by ED using monovalent selective ion exchange membranes and reported a recovery rate of 94.5% and an energy consumption index of only 1.9 W h/g Li^+^.^53^ Xie et al. applied a solar-assisted ED system for lithium recovery from spent lithium iron phosphate batteries. The results showed that the energy consumption for lithium extraction under light illumination was 12.90 W h/g Li, which was much lower than 16.20 W h/g Li for the process without light illumination. The membrane used in the ED cell, its characteristics, the composition of the feed solution, and the combination of ED with other techniques lead to differences in specific energy consumption.^64^

## 3 Conclusions

The effects of different parameters on Li^+^ removal by EDI were studied, and results showed that when 20 V was applied to the EDI stack, the Li^+^-containing solution was circulated at a stream rate of 2 L/h, and 99% of Li^+^ was eliminated from the solutions. The results also showed that the removal efficiency was proportional to the applied potential, and a decrease in the applied potential resulted in a reduction in the removal rate. The flow rate of the feed is another parameter that should be considered. When the stream rate of the Li-containing solution diminished, so did the elimination rate of Li^+^. The rate of removal of Li^+^ is affected by the concentration of Na_2_SO_4_ solution. When the amount of Na_2_SO_4_ in the solution was increased, the percentage of Li^+^ removal also increased. The coexistence of Na^+^ and K^+^ at low concentrations did not interfere with the removal rate of Li^+^. The mass transport coefficient of Li^+^ is calculated as 5.39 × 10^–4^ m/s under optimum conditions.

## 4 Materials and Methods

### 4.1 Chemicals

Li_2_CO_3_ (Merck) and HCl (Carlo Erba) were used to prepare the Li^+^ stock solution as described in ref (12). Na_2_SO_4_ (Analar) was used to prepare the electrolyte solution to circulate in the electrode chambers. NaCl, KCl, MgCl_2_·6H_2_O, Ba(NO_3_)_2_ and CaCl_2_·2H_2_O (Merck) salts were used to prepare the interfering ion-containing solutions.

### 4.2 Ion-Exchange Resins and Membranes

The strongly acidic cation exchange resin Purolite C145 and the strongly basic anion exchange resin Purolite A500plus are used together with the Selemion anion exchange membrane and the CEM to obtain a resin-filled central compartment. Before use, ion exchange membranes were conditioned with NaCl solution, as explained in ref (65). The characteristics of resins are summarized in Table S1 (Supporting Information file).

### 4.3 EDI Cell

The microflow EDI cell was used in the experiments which consists of two chambers, the central (dilute) anode and the cathode chamber. A stainless steel cathode and a dimensional stable anode were used as electrodes. The mixed-bed-resin-filled central compartment is shown in Figure 6, and the EDI experiment flowchart is shown in Figure S1. The experimental conditions are summarized in Table S2.

### 4.4 Lithium Analysis

The quantity of Li^+^ in samples was measured by a flame photometer (Jenway) as indicated in standard methods.^66^ 0.1 mg of Li/L can be detected after selecting the appropriate reading with the fine and coarse sensitivity controls.

## Supporting Information Available

Additional experimental details, materials, formulas, and methods, including the photograph of the experimental setup (PDF)
