# Supplementary material for: Utilization of Electrodeionization for Lithium Removal
Source: ACS Omega. 2023 May 10;8(20):17583–90. doi: 10.1021/acsomega.2c08095 (PMC10210215; doi:10.1021/acsomega.2c08095)
Supplement: Supplementary file 1 — ao2c08095_si_001.pdf [file ao2c08095_si_001.pdf]

The utilization of Electrodeionization for Lithium removal

Gülseren DEMIR

Chemistry Department, Faculty of Science, Ege University, Izmir, Türkiye

Ayşe Nur MERT

Chemistry Department, Faculty of Science, Ege University, Izmir, Türkiye

Özgür ARAR*

Chemistry Department, Faculty of Science, Ege University, Izmir, Türkiye

2.2 Ion-exchange resins and membranes

The anion-exchange resin was contacted with 2.0 M NaOH solution to convert it to OH-form, while cation exchange resin was contacted with 2.0 M HCl to convert it to H-form.

Table S1: Typical physical and chemical characteristics of ion-exchange resins.

**Resin**	**A500plus**	**C145**
**Polymer Structure**	Macroporous polystyrene crosslinked with divinylbenzene	Macroporous polystyrene crosslinked with divinylbenzene
**Functional Group**	Type I Quaternary Ammonium	Sulfonic Acid
**Total Capacity, eq/L**	1.15 (Cl- form)	1.5 (Na+ form)
**Temperature Limit, °C**	65 (OH- form)	120 (H+ form)

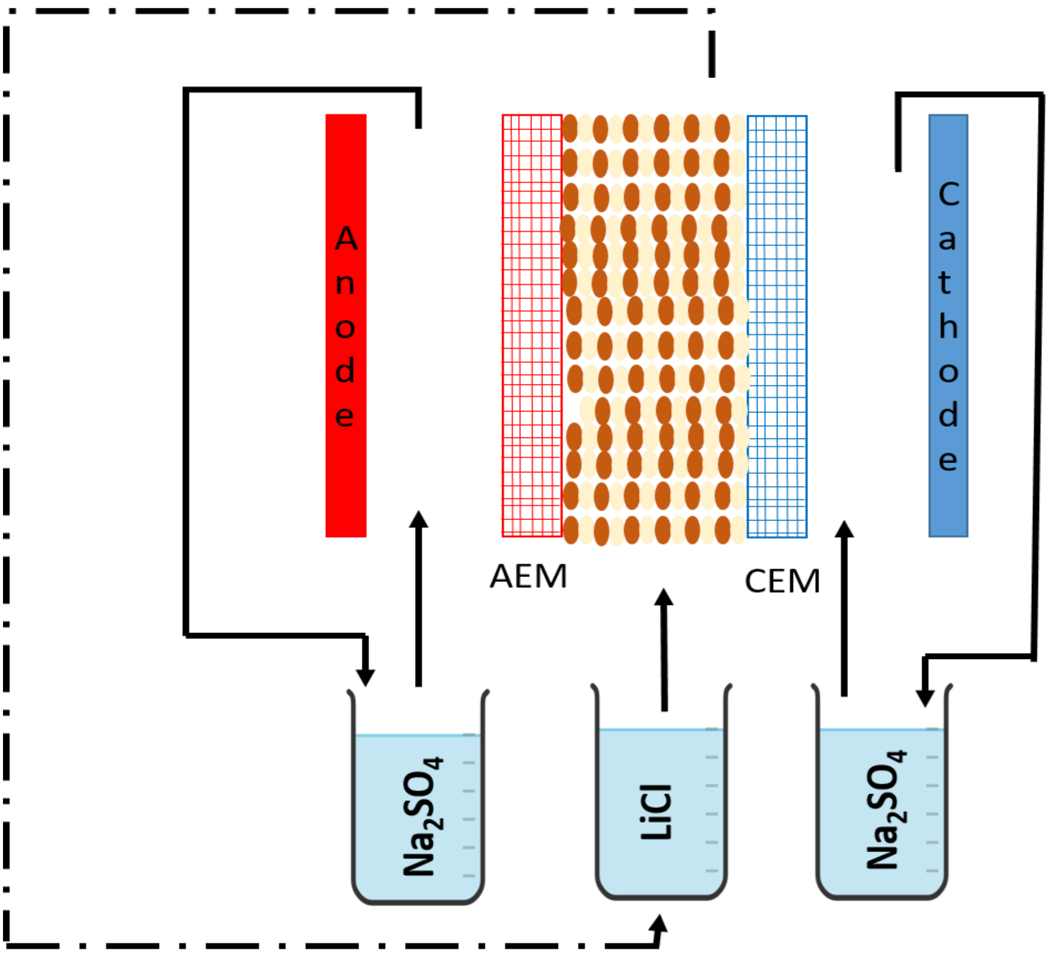

Figure S1: Illustration of the EDI stack (AME: Anion exchange membrane, CME: cation exchange membrane, amber (anion exchange), and brown (cation exchange) beads are ion exchange resins).

Table S2: Experimental conditions for each parametric test.

Experimental parameter	Applied Voltage (V)	The flow rate of Li-containing solution (L/h)	Li^+-concentration (mg-Li/L)	Na_2SO_4 concentration (M)	Na_2SO_4 flow rate (L/h)
**Applied Voltage**	10 – 30	2	5	0.01	6
**Feed flow rate**	20	1-3	5	0.01	6
**Na_2SO_4 concentration**	20	2	5	0.005 – 0.05	6

2.3 EDI cell.

The anion exchange resins (2.5 mL) and cation exchange resins (2.5 mL) were mixed in a beaker to form a mixed bed, and the central chamber of the cell was filled with the mixed bed configuration.

2.4 Preparation of Li^+ stock solution

A stock solution of Li^+ was prepared by dissolving appropriate amounts of Li_2CO_3 (Merck) salt. The weighed amount of Li_2CO_3 was transferred to a beaker containing pure water and then HCl was added to completely dissolve the solution. The solution was heated and swirled to drive out dissolved CO_2. The prepared solution was cooled to room temperature, then transferred to a volumetric flask and diluted to volume with water.

3.5 EDI Performance

The performance of EDI was examined by the calculation flux (J), mass transfer coefficient (k), and specific power consumption (SPC), respectively. The following equations (Eq. 1-3) are applied for such purpose, and the results are summarized in Table 2 of the main text [1–4]

$$J = (C_i - C_f)\frac{Q}{nA} \tag{1}$$

$$k = \frac{(C_i - C_f)}{C_i}\frac{Q}{nA} \tag{2}$$

$$SPC\ \left(\frac{Wh}{g}\right) = \frac{E\int_0^t Idt}{(C_i - C_f)M\,V_D} \tag{3}$$

C_i: Initial lithium concentration in the feed solution (mole/m^3)

C_f: Final lithium concentration in the feed solution (mole/m^3)

Q: Feed flow rate (m^3/s)

n: Number of cell pairs (1, in our case)

A: membrane area (m^2)

M molar mass of LiCl (42.394 g/mole)

V_D: The volume of the solution was fed to the central compartment (m^3)

The mass transfer coefficiecnt calclateions was carried out as follows.

In the experiment at 30 V, the initial concentration of Li+ was 4.75 mg/L (C_i = 0.684 mol/m^3) and at the end of the experiment it decreased to 0.06 mg/L (C_f= 0.0091 mol/m^3). The flow rate was 2 L/h (Q= 5.555x10^{-7} m^3/s). The membrane area is 3 cm x 3.4 cm = 10.2 cm^2 (A= 0.00102 m^2) and n= 1 for our cell. Put these values into equation 2

$$k = \frac{(0.684 - 0.0091)mol/m^3 x 5.555 \times 10^{-7} m^3/s}{0.684 mol/m^3\ x 1 x 0.00102 m^2} = 5.37x10 - 4\ m/s$$

References

(1) Dermentzis, K. Removal of Nickel from Electroplating Rinse Waters Using Electrostatic Shielding Electrodialysis/Electrodeionization. *J. Hazard. Mater.* **2010**, *173* (1–3), 647–652. https://doi.org/10.1016/j.jhazmat.2009.08.133.

(2) Dermentzis, K.; Christoforidis, A.; Papadopoulou, D.; Davidis, A. Ion and Ionic Current Sinks for Electrodeionization of Simulated Cadmium Plating Rinse Waters. *Environ. Prog. Sustain. Energy* **2011**, *30* (1), 37–43. https://doi.org/10.1002/ep.10438.

(3) Laktionov, E.; Dejean, E.; Sandeaux, J.; Sandeaux, R.; Gavach, C.; Pourcelly, G. Production of High Resistivity Water by Electrodialysis. Influence of Ion-Exchange Textiles as Conducting Spacers. *Sep. Sci. Technol.* **1999**, *34* (1), 69–84. https://doi.org/10.1081/SS-100100637.

(4) Lv, Y.; Yan, H.; Yang, B.; Wu, C.; Zhang, X.; Wang, X. Bipolar Membrane Electrodialysis for the Recycling of Ammonium Chloride Wastewater: Membrane Selection and Process Optimization. *Chem. Eng. Res. Des.* **2018**, *138*, 105–115. https://doi.org/10.1016/j.cherd.2018.08.014.
